# Systematic review: non-adherence and non-persistence in intravitreal treatment

**DOI:** 10.1007/s00417-020-04798-2

**Published:** 2020-06-22

**Authors:** Christoph Ehlken, Focke Ziemssen, Nicole Eter, Ines Lanzl, Hakan Kaymak, Albrecht Lommatzsch, Alexander K. Schuster

**Affiliations:** 1 Klinik für Ophthalmologie des Universitätsklinikums Schleswig-Holsteins, Campus Kiel, Kiel, Germany; 2 Center for Ophthalmology, Eberhard Karl University, Tübingen, Germany; 3 Klinik für Augenheilkunde, Universitätsklinikum Münster, Münster, Germany; 4 Chiemsee Augen Tagesklinik, Prien am Chiemsee, Germany; 5 Makula-Netzhaut-Zentrum, Düsseldorf-Oberkassel, Germany; 6 Augenzentrum am St. Franziskus-Hospital Münster, Münster, Germany; 7 Augenklinik und Poliklinik, Universitätsmedizin Mainz, Mainz, Germany

**Keywords:** VEGF, Adherence, Persistence, AMD, DME, Intravitreal

## Abstract

**Purpose:**

Intravitreal injection of VEGF inhibitors has become the standard of care for different macular diseases within the last years resulting in improved visual outcomes. Under real-life conditions, however, the necessity for frequent retreatments and reexaminations poses a burden for patients and treatment centers. Non-adherence and non-persistence to intravitreal treatment may lead to inferior clinical outcomes, and knowledge of contributing factors is crucial to improve adherence. This systematic review analyzes current literature for potential factors involved in non-adherence and non-persistence.

**Methods:**

A systematic search was conducted in PubMed and Embase including three different aspects of intravitreal injection therapy: (1) diseases with intravitreal injections as treatment, (2) intravitreal injection, and (3) aspects of therapy adherence or therapy persistence. Data from identified quantitative studies were further extracted and grouped according to WHO criteria (condition, socio-economy, therapy, patient, and health system). The methodological quality of identified studies was graded. Identified qualitative studies (i.e., interviews) were descriptively analyzed and their findings narratively reported.

**Results:**

Twenty-four publications were included. In 16 of those publications, a quantitative data analysis was conducted, analyzing factors associated with non-adherence. Worse visual acuity at baseline and unfavorable development of visual acuity, higher age, and greater distance to the treatment center were associated with non-adherence, while there was inconsistent evidence for an association of comorbidity. In qualitative studies, high follow-up/treatment burden, fear and anxiety, disappointed patient expectations, and lack of motivation to continue treatment were reported as reasons for non-persistence.

**Conclusions:**

Knowledge of potential barriers in IVT treatment may improve adherence and potentially clinical results. Improvements can be achieved particularly in the healthcare complex (organizational improvements) and the “patient” complex by establishing realistic expectations. Recurrent education of the patient may be necessary.

**Electronic supplementary material:**

The online version of this article (10.1007/s00417-020-04798-2) contains supplementary material, which is available to authorized users.

## Introduction

Intravitreal injection of VEGF inhibitors has become the standard of care for different retinal and chorio-retinal disorders within the last years. They were introduced as a therapeutic option for patients with neovascular age-related macular degeneration (nAMD), but their use was soon extended to diabetic macular edema, to macular edema secondary to retinal vein occlusion (RVO), and to myopic choroidal neovascularization (mCNV) or CNV of other causes. These retinal diseases are found especially in older age, and thus their frequency is increasing further with the demographic changes. The number of patients diagnosed with nAMD alone has been estimated to rise to up to 9 million globally in 2040 [1]. Personnel as well as cost requirements are not only a challenge for healthcare systems. The number of treatments and follow-ups is just as much a personal burden for those affected as their relatives in chronic diseases [2]. The pivotal randomized trials were designed to demonstrate the efficacy of anti-VEGF with fixed or clearly defined retreatment schemes within a relatively short study duration. Further post-authorization studies suggest that similar efficacy of “as-needed” [3, 4] and “treat-and-extend” protocols [5] may exist, reducing the number of treatments and/or visits. However, application in clinical routine is at significantly higher risk of being disturbed by external factors such as logistical and financial burdens [6]. As a consequence, patients with nAMD receive fewer anti-VEGF intravitreal injections (IVT) in the real-world setting compared with clinical trials. The low annual number of injections has been reported to have immediate impact on the visual outcome [7, 8]; the regularity of the scheduling of treatments or control visits is of vital importance in order to avoid undertreatment [9].

### Non-adherence and non-persistence

Adherence in long-term therapy, as defined by the World Health Organization (WHO), describes “the extent to which a person’s behavior – taking medication, following a diet, and/or executing lifestyle changes – corresponds with agreed recommendations from a health care provider” [10]. Consequently, *non-adherence* to intravitreal therapy can be defined as the extent of deviation from the previously planned retreatment intervals, including the unplanned extension of the control regime [11]. While oral therapies and eye drops require elaborate measures to assess the actual non-adherence at home [12], the attending ophthalmologist can usually determine precisely the number and intervals of IVTs as well as control examinations. Some patients might be particularly prone to missing appointments, therapy breaks, and suspension of retreatment despite activity [13–17]. The danger of a delay is not only present at the beginning of the treatment [18] but also during the follow-up, e.g., due to logistical factors such as summer vacations [19].

*Non-persistence* is the final withdrawal from the treatment regime. It can be related to patient-centered factors such as disappointment resulting from unmet expectations, comorbidities, or transport problems [20, 21]. The failure to notice improvement during monotonous repetition, the burden placed on relatives or carers, and the inconveniences, such as irritations of the eye surface, may prompt people to consider whether it is worth the effort in view of their life expectancy [2]. The frequency of the problem as well as the consequences can vary considerably between the diseases. For example, a stop of treatment can quickly endanger the eyesight in nAMD [22] and proliferative diabetic retinopathy [23].

### WHO action and prioritization of non-adherence

The WHO has identified poor adherence to treatment of chronic diseases as a worldwide problem of striking magnitude. The consequences of non-adherence to long-term therapies are worse health outcomes and increased healthcare costs. The WHO report on “Adherence to long-term therapies” includes chronic diseases, such as tuberculosis, HIV/AIDS, depression, epilepsy, substance dependence (exemplified by smoking cessation) as well as hypertension, asthma, and palliative care for cancer [10]. However, retinal diseases have not been included so far. For the included chronic diseases, utilization and health outcomes are strictly monitored and described in published reviews by the WHO. Studies consistently find significant cost savings and increases in the effectiveness of health interventions that are attributable to low-cost interventions for improving adherence.

**Fig. 1:**
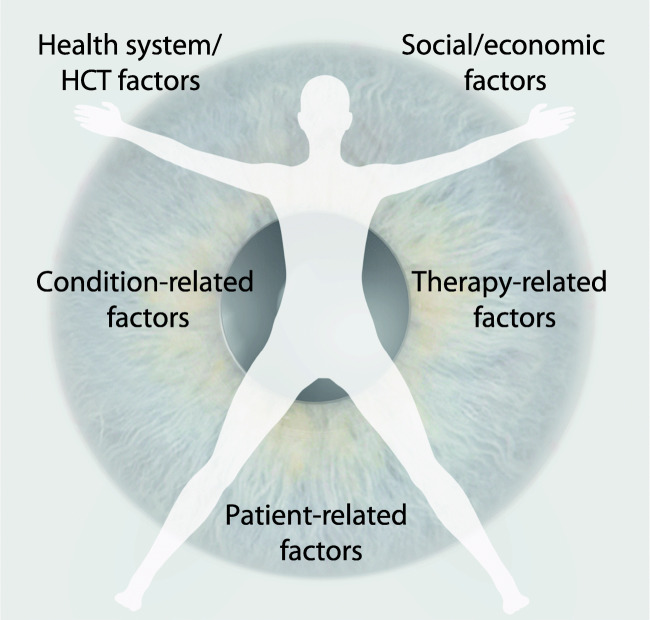
The five dimensions of adherence according to the WHO [10]

The WHO identifies five dimensions that have a significant influence on adherence to long-term therapies (Fig. 1).

In order to improve adherence, three topics need to be simultaneously addressed as follows: knowledge (information on adherence), thinking (the clinical decision-making process), and action (behavioral tools for healthcare professionals) [24].

### Non-adherence as an underexposed topic in ocular diseases

The absence of any meta-analysis is in stark contrast to the high relevance of the required treatment exposure. The necessary systematic review promises to identify relevant factors that need to be considered specifically for IVT. Without a system that addresses the determinants of non-adherence, advances in biomedical drugs will fail to realize their potential to reduce the burden of chronic eye diseases [25].

The aim of this systematic literature research is to analyze the current evidence of factors associated with non-adherence in order to identify possible options for how current care can be improved for patients who are suffering from chronic retinal diseases and being treated with intravitreal drugs.

## Methods

### Systematic literature review

A systematic literature review, which included the databases Medline (via PubMed) and Embase, was conducted in December 2018 in order to identify articles analyzing therapy adherence to IVT. The search frame consisted of three different combined aspects: (1) diseases with potential treatment using IVTs such as age-related macular degeneration, diabetic macular edema, and retinal vein occlusion; (2) intravitreal injection; and (3) aspects of therapy adherence. Aspects of therapy adherence included therapy adherence (following recommended intervals and undergoing intravitreal injection or control examinations), therapy persistence (continuing with intravitreal treatment or regular controls over time), and combinations of those. The exact search terms are presented in supplementary information 1 (Suppl. 1).

The abstracts of all identified references were screened by two independent investigators (Ehlken and Schuster). Inclusion criteria were defined as follows: (1) observational studies analyzing patients with age-related macular degeneration, diabetic macular edema, or retinal vein occlusion; (2) patients receiving intravitreal injection (without specification on medication); and (3) data on therapy non-adherence (NA) or interview on factors influencing adherence/persistence. The full text of all potentially relevant publications were obtained and screened. Authors of potentially relevant conference abstracts were contacted twice and asked to provide further data. Randomized controlled trials were excluded due to potential selection bias and incentives to complete the study. Differences of selection between reviewers were resolved in consensus meetings.

Data extraction was performed separately for studies using either quantitative or qualitative approaches. Data were further extracted and grouped according to WHO criteria, namely, condition (i.e., type of diagnosis, state of disease at first diagnosis and under therapy, fibrosis and hemorrhage, visual acuity), socio-economy (i.e., age, education, income), therapy (e.g., complexity of treatment, treatment protocols, efficacy of treatment, adverse effects), patient (e.g., patient knowledge of disease and treatment, resources, motivation, expectations), and health system (i.e., availability and accessibility of treatment capacity, relationship between attending physician and patient).

### Quality assessment

The methodological quality of identified studies was graded using the Study Quality Assessment Tool of the National Institute of Health for observational cohort and cross-sectional studies [26]. This quality assessment included 14 items with respect to research question, definition of study population, participation rate of eligible persons, time and context of study recruitment, exposure of interest measured prior to outcome, sufficient timeframe, different levels of the exposure, definition of exposure measure, assessment of exposure more than once over time, definition of outcome measure, outcome assessor blinded to exposure status of study participant, and adjustment for potential confounding variables. The items “sample size justification” and “loss to follow-up” were discarded, as sample size calculation is usually not performed in real-life studies and non-adherence interferes with the loss to follow-up. Differences in quality grading between reviewers were discussed in a consensus meeting. Overall quality rating such as good, fair, or poor was conducted according to the recommendations.

Identified qualitative studies (i.e., interviews) were descriptively analyzed, and their findings are narratively reported.

## Results

### Literature research

#### Eligible studies

**Fig. 2:**
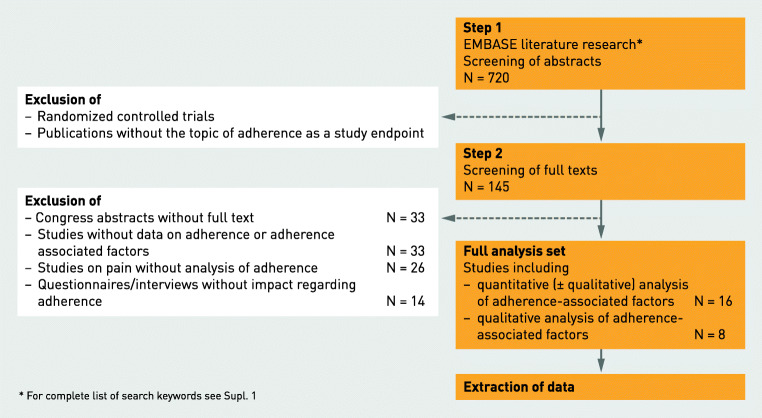
Flow chart literature research

The systematic literature research identified 720 publications meeting the prespecified screening criteria. All abstracts were scrutinized by two reviewers, and 145 were selected for an in-depth full-text screening. After careful consideration, 24 publications met the eligibility criteria and were included in data analysis. In 16 of those 24 publications, a quantitative data analysis was conducted, evaluating factors associated with non-adherence (Fig. 2). Eleven of these 16 articles included additional data from interviews or questionnaires addressing factors contributing to non-adherence in those patients (qualitative analysis). Eight studies reported qualitative data on patient interviews of treated but not necessarily non-adherent patients, addressing possible attributable factors. Details regarding the included studies are provided in Tables 1 and 2. Studies on pain and discomfort were not included in the main analysis [27–45]. However, details and main conclusions of these studies are provided in the supplementary material.

**Table 1:** Characteristics of included studies

First author	Country	Time span	Study type	Mono-/multicenter	Disease	Data acquisition	Mean follow-up (years)	Number of patients	Definition non-adherence
Atchison [46]	USA	Unclear	Retrospective cohort	Mono	nAMD	Chart review^a^	≤ 4	556	“Interrupted treatment cycles”
Boulanger-Scemama [13]	France	2006–unclear	Retrospective cohort	Mono	nAMD	Chart review, questionnaires^a^	5	201	No follow-up visit for at least 6 months
Curtis [47]	USA	2004–2009	Retrospective cohort	Multi	nAMD	Health claim data	3	284,380	“Discontinuation”: absence of treatment for 12 months
Droege [14]	Germany	Unclear	Cross-sectional cohort	Mono	nAMD	Chart review, interview^a^	1.8	95	“Discontinuation”: no further visits; “adherence” = time to discontinuation
Ehlken [17]	Germany	2011–2015	Retrospective cohort	Mono	nAMD, DME, BRVO	Chart review	1	708	Unintended gap > 56 days between IVT and OCTs
Ehlken [48]	Germany	2011–2012	Phase IV (retrospective/prospective)	Multi	nAMD	Chart review, phase IV trial	≤ 2 retrosp.1 prosp.	480	“Non-adherence”: no OCT/treatment for 6 weeks; “non-persistence” = > 3 months
Gillies [49]	Australia	2013–2016	Retrospective registry	Multi	nAMD	Registry^a^	1	394 (eyes)	“Non-completers”: less than 12 months of follow-up
Heimes [50]	Germany	2010–unclear	Retrospective consecutive cohort	Mono	nAMD	Chart review, interview^a^	2.4	191	No control within 6 months or appointment missed for more than 2 weeks
Krüger Falk [51]	Denmark	2007–2011	Retrospective cohort	Mono	nAMD	Chart review^a^	1.3–4	855 (eyes)	“Stopped treatment”
McGrath [52]	Australia	2008–2010	Retrospective case control	Mono	nAMD, others	Chart review; interview^a^	3	250	“Dropouts”: failed to attend visits for > 6 months
Nunes [53]	Brazil	2006–2008	Retrospective consecutive cohort	Mono	nAMD	Chart review, interviews^a^	2	82	No control within 3 months
Oishi [54]	Japan	2008–2011	Retrospective cohort	Mono	nAMD	Chart review	1	87	“Cessation”: no injection for more than 6 months
Polat [15]	Turkey	2009–2011^b^	Retrospective cohort	Mono	nAMD	Chart review, interviews^a^	1	314	No “load-up”, or no continuation during first year
Weiss [16]	Germany	2011–2015	Retrospective cohort	Mono	nAMD, DME	Clinical registry^a^	DME 2.5 AMD 1.9	245	Difference > 14 days to intended appointment; >100 days = break-off
Westborg [55]	Sweden	2013–2015^b^	Retrospective registry	Multi	nAMD	Registry	1	932	No treatment/control visit during months 10–14
Ziemssen [56]	Germany	2009–2011	Phase IV	Multi	nAMD	Phase IV trial^a,c^	2	420	Stop of treatment

^a^Including interviews or questionnaires addressing reasons for non-adherence in non-adherent patients

^b^Initial diagnosis

^c^Presentation of subgroup of patients from Germany

**Table 2:** Quality rating of included quantitative studies

Publication	1. Research objective clearly stated?	2. Study population clearly specified and defined?	3. Participation rate of eligible persons ≥ 50%?	4. Subjects recruited from same populations? Inclusion criteria prespecified?	6. Exposure of interest measured prior to outcomes?	7. Time frame sufficient?	8. Different levels of exposure examined?	9. Exposure measures clearly defined, valid, reliable, implemented consistently across study participants?	10. Exposures assessed more than once over time?	11. Outcome measures clearly defined, valid, reliable, implemented consistently across study participants?	12. Outcome assessors blinded to exposure?	14. Key potential confounding variables measured, adjusted statistically?	Quality rating (good/fair/poor)
Atchison [46]	Yes	Yes	Unclear	Yes	No	Yes	Yes	No	No	Yes	Unclear	No	Poor
Boulanger-Scemama [13]	Yes	Yes	Yes	Yes	No	Yes	Yes	Yes	No	Yes	No	Yes	Good
Curtis [47]	Yes	Yes	Yes	Yes	No	Yes	Yes	Yes	No	Yes	No	Yes	Fair
Droege [14]	Yes	Yes	Unclear	Yes	No	Yes	Yes	Yes	No	Yes	No	Yes	Good
Ehlken [17]	Yes	Yes	Unclear	Yes	Yes	Yes	Yes	Yes	No	Yes	Yes	Yes	Good
Gillies [49]	Yes	Yes	Unclear	Yes	Yes	Yes	Yes	No	No	Unclear	Unclear	No	Fair
Heimes [50]	Yes	Yes	Yes	Yes	Unclear	Yes	in part^1^	In part^1^	No	Yes	No	No	Fair
Krüger Falk [51]	Yes	Yes	Yes	Yes	Unclear	Yes	Unclear	No	No	Yes	Unclear	No	Fair
McGrath [52]	Yes	Yes	Yes	Yes	Yes	Yes	Yes	Yes	No	Yes	Unclear	Yes	Good
Nunes [53]	Yes	Yes	Yes	Yes	No	Yes	No	No	No	Yes	No	No	Poor
Oishi [54]	Yes	Yes	Unclear	No	Unclear	Yes	Yes	Yes	No	Yes	Unclear	No	Fair
Polat [15]	Yes	Yes	Unclear	Yes	Unclear	Yes	Yes	Unclear	No	Yes	Unclear	Yes	Fair
Weiss [16]	Yes	Yes	Yes	Yes	Unclear	Yes	No	Not applicable	Yes^2^	Yes	Unclear	Yes	Good
Westborg [55]	Yes	Yes	Unclear	Yes	Yes	Yes	Yes	Yes	Unclear	Yes	Yes	No^3^	Fair
Ziemssen [56]	Yes	Yes	Unclear	Yes	Unclear	Yes	No	Unclear	No	Yes	Unclear	No	Fair
Ehlken [48]	Yes	Yes	Yes	Yes	Yes	Yes	Yes	Yes	Yes	Yes	Yes	Yes	Good

^1^Different levels were defined for distance evaluation

^2^Entries in database. If no reason for non-adherence was stated in medical charts/database, one phone call was made

^3^Register data and data imputation methods used. Index for comorbidities used

#### Evaluation of methodological quality of identified studies

The methodological quality of factors associated with NA was evaluated in the 16 studies listed in Table 2. Six studies received the quality rating “good,” and eight studies were rated as “fair” quality and two as “poor” quality, respectively. The most common reasons leading to a lower quality score were the lack of analyzing key potential confounding factors in the studies (item 14) and the lack of definition of exposure measures (item 9).

### Factors associated with non-adherence

#### WHO complexes

**Fig. 3:**
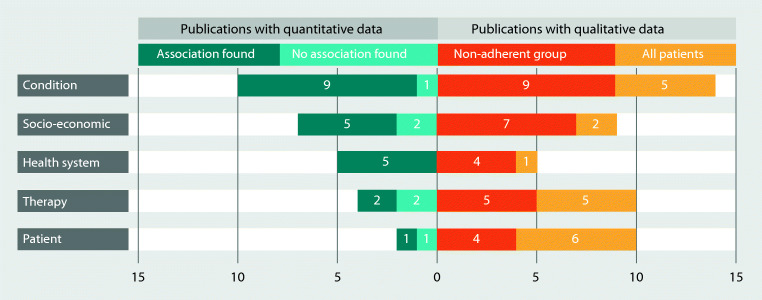
Number of publications reporting on factors classifies by WHO dimensions

Data availability differs between the different WHO dimensions. The frequency of reported associated complexes is displayed in Fig. 3, and the corresponding studies and attributable factors are shown in Table 3.

**Table 3:** Studies with qualitative/quantitative analysis in regard of WHO complexes

		Quantitative analysis		Qualitative analysis	
*WHO complex*	*Factor*	*Association found*	*No association found*	*Non-adherent patients*	*All patients*
Condition	Comorbidity	Westborg et al. [55]	McGrath et al. [52]^1^, Polat et al. [15]	Droege et al. [14], Gillies et al. [49], Heimes et al. [50], Krüger Falk et al. [51], Nunes et al. [53], Polat et al. [15], Weiss et al. [16]	Polat et al. [15]^2^
	Fellow eye	Ehlken et al. [48]^3^	Polat et al. [15]		
	Lower VA at baseline	Ehlken et al. [17], Oishi et al. [54], Polat et al. [15], Westborg et al. [55]			Baxter et al. [57]
	VA change (subj. or obj.)	Oishi et al. [54], Polat et al. [15], Weiss et al. [16], Ziemssen et al. [56]		Boulanger-Scemama et al. [13], Gillies et al. [49], McGrath et al. [52]^4^, Nunes et al. [53], Polat et al. [15], Weiss et al. [16], Westborg et al. [55]	Boyle et al. [2], Sii et al. [58], Kostadinov et al. [59], Mueller et al. [20]
Health system	Center-dependent	Ehlken et al. [17], Heimes et al. [50], Westborg et al. [55], Ehlken et al. [48]		Krüger Falk et al. [51], Nunes et al. [53], Polat et al. [15], Weiss et al. [16]	
Patient	Anxiety, fear		Droege et al. [14]^5^	Polat et al. [15]	Boyle et al. [2], Droege et al. [60], Müller et al. [61], Senra et al. [62], Sii et al. [58], Kostadinov et al. [59]
	Motivation, knowledge, expectations			Droege et al. [14], Krüger Falk et al. [51], Weiss et al. [16], Westborg et al. [55]	Boyle et al. [2], Müller et al. [61], Sii et al. [58]
Socio-economic	Education	Polat et al. [15]			
	Age	Boulanger-Scemama et al. [13], Ehlken et al. [17], Oishi et al. [54]^6^, Polat et al. [15], Ehlken et al. [48]			
	Travel, distance to treatment	Boulanger-Scemama et al. [13], McGrath et al. [52], Polat et al. [15]		Boulanger-Scemama et al. [13], Heimes et al. [50], Nunes et al. [53], Polat et al. [15], Weiss et al. [16]	Droege et al. [60]
	Financial burden, reimbursement			Boulanger-Scemama et al. [13], McGrath et al. [52], Polat et al. [15], Weiss et al. [16], Ziemssen et al. [56]	Kostadinov et al. [59]
Therapy	Type of drug	Westborg et al. [55]^7^	Curtis et al. [47]^7^, Gillies et al. [49]^7^		
	Fear of adverse events			Atchison et al. [46], Krüger Falk et al. [51], Weiss et al. [16], Westborg et al. [55], Ziemssen et al. [56]	Boyle et al. [2], Droege et al. [60], Kostadinov et al. [59], Mueller et al. [20]
	Treatment regimen, follow-up burden	Ehlken et al. [48]		Boulanger-Scemama et al. [13], Krüger Falk et al. [51]	Droege et al. [60], Müller et al. [61], Senra et al. [62]

^1^Comorbidity was associated with less non-adherence in this study from Australia

^2^Comorbidity identified as relevant in interviews, but not identified as factor in statistical analysis

^3^Treatment-dependent AMD in fellow eye associated with higher risk for NA

^4^Patients with a subjective good vision had a higher risk of stating that IVT is not needed despite recommendation from their attending ophthalmologist

^5^No correlation with number of injections was found in this study

^6^In this study, lower age was associated with non-adherence, although this was not true in bivariate analysis

^7^[55]: More NA in patients treated with ranibizumab than aflibercept. [47, 49]: No difference between ranibizumab and bevacizumab or aflibercept and ranibizumab

Most evidence was found for the dimension “condition” (e.g., diagnosis, state of disease at first diagnosis and while under therapy), in both the quantitative analysis and in-patient interviews. Considerable evidence was found for the dimensions “socio-economy” (e.g., age, education, wealth) and “health system” (availability and accessibility of medication and treatment capacity, relationship between attending physician and patient, availability of information). Descriptive analysis of quantitative studies only showed limited evidence in the dimensions “therapy” (e.g., complexity of treatment, treatment protocols, efficacy of treatment, adverse effects) and “patient” (e.g., patient knowledge of disease and treatment, resources, motivation, expectations), although in studies with patients’ interviews, those aspects were repeatedly reported.

#### Factors associated with non-adherence

Studies on associated factors with NA either in quantitative studies or patient interviews are indexed in Table 3. In the following paragraphs, evidence is reported for nAMD, unless otherwise indicated. Methods of statistical analysis differed between studies so that different endpoints may be reported for individual factors. The most prominent factors will be described below.

### Dimension “condition”

#### Association of visual acuity and NA

Quantitative studies frequently and consistently identified an association of *worse visual acuity at baseline* and *unfavorable development of VA* with the occurrence of NA. Odds ratios for *worse VA at baseline* ranged from 1.42 (Weiss et al., for BCVA ≤ 20/60, *p* < 0.001) [16], over 2.37-fold (Ehlken et al., for BCVA at baseline ≥ 0.4 logMAR, *p* = 0.048) [17] to 8.1-fold (Oishi et al., no cut-off given) [54]. Spearman’s correlation ranged from *r* = 0.38 (Oishi et al.) [54] to *r* = 0.43 (Polat et al.) [15]. For *unfavorable development of VA****,*** Spearman’s correlation was calculated with *r* = 0.22 (Polat et al.) [15]. VA was significantly worse in non-adherent patient groups (Oishi et al.) [54]. In addition, *unfavorable development of VA* was mentioned in multiple interviews by patients in the *non-adherent* subgroup as well as the total cohort.

#### Comorbidity

There is inconsistent evidence for an association of *comorbidity* with NA in quantitative studies. Westborg et al. calculated an OR of 1.27 (*p* = 0.001) for NA for patients with significant comorbidity, defined as a Charlson Comorbidity Index ≥ 1 [55]. Polat et al. found no statistically significant association (*p* = 0.87) [15], and McGrath et al. even described a higher rate of NA in otherwise healthy patients in an Australian cohort (48.7% NA vs. 31.3%, *p* = 0.04) [52]. In patient interviews of non-adherent patients, however, comorbidity was commonly stated as a factor contributing to NA, even in the same study from Polat et al.

### Dimension “socio-economy”

#### Higher age

*Higher age* was frequently identified as a factor associated with NA. Ehlken et al. described a positive association of higher age with an OR of 1.04/year (*p* = 0.013) in a mono-center study [17] and an OR of 1.05/year (*p* = 0.03) in the PONS multicenter trial [48]. In a study by Boulanger-Scemama [13], higher age was significantly associated with loss to follow-up but not with NA. However, Oishi et al. report that lower age was associated with a higher risk for NA in a Japanese cohort (OR 0.94, 95%-confidence interval 0.89–0.99) [54].

#### Travel and distance to treatment

A *greater distance to the treatment center* was also identified as a potential contributing factor to NA. Boulanger-Scemama et al. found a significant correlation between a greater distance and loss to follow-up (*p* = 0.007), and distance was mentioned as a main contributing factor in interviews of non-adherent patients in this setting (30/58 patients), more often than any other reason [13]. In an Australian cohort, a journey of > 100 km was also associated with a higher dropout (50% vs. 28%). In addition, travel and greater distance were mentioned by patients in multiple interview studies [52].

#### Ethnicity

Curtis et al. reported that nonwhite patients were less likely than white patients to receive an anti-VEGF agent (OR 0.77, 95%-confidence interval 0.75–0.79) [47]. However, the authors discussed that several reasons may be attributable. They stated that unusual manifestations of the underlying disease (such as polypoidal choroidal vasculopathy in nAMD) may have been more common in nonwhite people and treatment recommendations were not established for these subtypes at the time of the study. In addition, the cost of anti-VEGF treatment, repeat access to clinics, and perceived risks of therapy may also have played a role. There were no studies that quantified the effect of ethnicity on adherence.

#### Financial burden

There was no study investigating problems with *reimbursement or financial burden* in any quantitative study. However, financial burden was listed by non-adherent patients in multiple studies from different countries.

### Dimension “health system”

#### Center dependency and NA

The PONS study identified *treatment at one center* as opposed to a system of referral and treating physicians as well as *treatment center experience* as protecting factors for NA (OR 0.33, *p* = 0.001, and OR 0.89, *p* = 0.044, respectively) [48]. Heimes et al. reported an increased adherence in patients treated in a single center with an intensified information and education system for patients after 12 months compared with regular treatment in other sites being electronically connected in a network [50]. Ehlken et al. described a significant deficiency of timely organization of retreatments in a mono-center cohort (approximately 20% of non-adherence according to a definition of retreatment/examination every 8 weeks) [17].

Problems with the treatment center (either with the attending physician or organizational structures) were confirmed in patient interviews in different studies, although those studies were rare.

#### Dimension “therapy“

There is a discrepancy in the available data concerning therapy-related issues between evidence from quantitative studies and patient interviews.

#### Treatment regimen and follow-up burden

There is only scant evidence regarding *treatment regimen* and NA in the identified studies. One study described fixed appointments at the treating center as being associated with a lower risk of NA (OR 0.45, *p* = 0.008), compared with referrals on demand by a referring ophthalmologist [48].

In patient interviews, a *high follow-up burden* was stated repeatedly, e.g., in the studies by Boulanger-Scemama (14/58 = 24.1% of NA cases) [13] and Krüger-Falk (3/36 = 8.3%, though no reasons were provided by 26/36 patients) [51]. In interviews of patients treated with IVT, more than 70% of patients judged the high treatment burden to be a possible barrier [60].

#### Adverse events

No study confirmed *adverse events* as attributable to NA in statistical analysis. However, in patient interviews, occurrence and fear of adverse events were repeatedly reported by non-adherent [16, 46, 51, 55, 56] and exposed patients [2, 14, 20, 59].

#### Type of anti-VEGF drug

One study from Sweden found a higher risk for NA in patients treated with ranibizumab compared with aflibercept (OR 1.45, *p* < 0.001) [55]. Two studies found no differences in NA between patients treated with bevacizumab and ranibizumab [47] or ranibizumab and aflibercept [49]. There was no report from patient interviews with regard to this aspect.

#### Dimension “patient”

In the dimension “therapy,” patient-associated factors were rarely addressed as associated factors in quantitative analysis. In patient interviews, however, factors such as *anxiety* or *fear* as well as *disease knowledge* and *motivation* were listed.

Fear or anxiety (29.6%) and lack of belief in treatment efficacy (21.6%) were stated most predominantly by non-adherent patients in a study from Turkey [15]. Fear and anxiety were also repeatedly reported as potential barriers in treated patients [2, 14, 58, 59, 61, 62].

Disappointment due to *unmet patient expectations* and *lack of motivation* to continue treatment were found in non-adherent patients in multiple studies [14, 16, 51, 55], although underlying causes for a patient’s desire to stop treatment were often not elucidated. Droege et al. described that anxiety was common in their study group (> 60%), but the numbers of injections were similar in patients with and without anxiety [14].

## Discussion

The WHO has defined non-adherence as a major potential threat in the care of chronic diseases [10]. However, eye diseases have not been specifically included in the current WHO analyses. This systematic review discusses factors involved in non-adherence and non-persistence in the treatment with intravitreal injections under real-life conditions. In the extracted publications, definitions of non-adherence and non-persistence differ depending on the study design, and similar endpoints may be called non-adherence or non-persistence in different publications. However, the high proportion of non-adherent and non-persistent patients with intravitreal injection therapy is a major limitation of this therapeutic option under real-life conditions, leading to inferior results in visual function [9, 17, 56]. Thus, identification of risk factors for non-adherence is pertinent, and knowledge of these factors may contribute to an improvement in the care of patients in need of IVT therapy. While awareness of modifiable factors that can be addressed in daily clinical work may be of special importance, knowledge of unmodifiable factors may also be valuable to better advise the individual patient.

### Unrealistic expectations may precipitate non-adherence

In this review, factors were grouped according to the five dimensions of adherence according to the WHO. Most evidence was found for the dimension “condition,” showing that *worse visual acuity* at baseline and *unfavorable development of visual acuity* were associated with non-adherence. Evidence for the factor “unfavorable development of visual acuity” was found in both the quantitative and qualitative analysis. This is intriguing since non-adherence itself may lead to inferior VA results and thus further precipitate non-adherence. Furthermore, the high rate of disappointment regarding the development of VA may be caused by unrealistic expectations, e.g., illustrated by the results of the interviews in the PONS study [20, 48]: Although many patients were aware of the need for repeated examinations and treatments, the majority was not aware of the chronic nature of nAMD and hoped to gain visual acuity during the course of the treatment. This emphasizes the need to establish realistic expectations, and it underlines the importance of a sensible education by the care providers (physicians and nurses).

### Differences between qualitative and quantitative analysis

There was no consistent association with *comorbidities*, and the disparity between quantitative analysis (association only in one study [55] and no association in two studies) and qualitative analysis (mentioned in 7 studies, Tab. 3) is noticeable. Non-adherent patients repeatedly listed comorbidity as a major contributing factor. The design of the majority of the studies was retrospective, and thus the disparity between them may be attributable to reporting bias or insufficient documentation, as well as differences in the definition. Reduced mobility, for example, with ensuing difficulties in attending regular appointments, may be regarded as an independent factor as well as the consequence of systemic comorbidity.

Considerable evidence was found for the dimension “socio-economy”: Higher age was associated with lower adherence, and financial burden was reported in several qualitative studies as a cause of non-adherence. Parameters of the “health system” (availability of and accessibility to medication and treatment capacity, relationship between attending physician and patient, availability of information) were repeatedly described in the qualitative analysis. Descriptive analysis of quantitative studies only showed limited evidence in the dimensions “therapy” (e.g., treatment protocols) and “patient” (e.g., patient knowledge of disease and treatment). In qualitative studies, *high follow-up burden* and *high treatment burden* were repeatedly cited as factors for non-adherence.

### Particular barriers in intravitreal therapy compared with other chronic diseases

Although the included studies identified a variety of factors affecting non-adherence, only few of them are modifiable. In addition, some obvious factors encountered in clinical everyday life, such as patient accompaniment to therapy, were not addressed in studies at all. These factors are not directly conferrable to identified risk factors for low therapy adherence in other chronic diseases such as arterial hypertension [63] or dyslipidemia [64]. Due to the particular nature of the treatment, which includes repetitive surgical injections into the eye, it may be rather compared with treatments such as subcutaneous depot injection, although the psychological tension experienced by the patients and the organizational requirements (i.e., operating theater in some countries) are different and presumably cause greater strain. In this analysis, pain and discomfort have not been proven to be relevant risk factors, but studies focusing on pain and possible approaches to prevent discomfort during the injection procedure were not specifically included. While multiple studies concerning pain and discomfort have been published, these rarely address its effect on adherence (see Suppl. 2) [65–67].

### Addressing modifiable and unmodifiable risk factors in everyday practice

The literature research identified both modifiable and unmodifiable risk factors for non-adherence. Modifiable factors were in the “health system” dimension (such as center-dependent risk factors, including organization, availability of short-term appointments, or phone service) and the “patient” dimension (such as anxiety or fear, patient knowledge, and motivation). Addressing modifiable factors seems a feasible approach to improve adherence. Improving organizational processes may considerably reduce center-associated barriers. However, this may require considerable effort and reallocation of resources for the treatment center. For example, establishing a better level of accessibility via telephone may require additional personnel and thus may be limited by financial considerations.

In the “therapy” dimension, the design and complexity of therapy protocols were commonly raised by patients. For the average patient, treatment protocols with a better ability to plan appointments and reduce examination or control visits (such as fixed or TAE protocols) may be advantageous compared to PRN. However, individual patients may prefer different treatment regimes, and this places additional burdens on the organizational endeavors of the treatment centers. Treatment in one treatment center (as opposed to a referral system) is preferred by patients, and patients preferred a “one-stop clinic” (examination and injection on the same day) over a referral system [57]. Treatment at one center was associated with less non-adherence in a German study [48]. However, treatment networks offering the possibility of direct data exchange and a cooperative approach may provide a high level of patient satisfaction and good clinical results [68, 69].

Many of the identified risk factors, such as age, involvement of the fellow eye, comorbidity, general education, or visual acuity at baseline, are unmodifiable, and a direct approach for improvement is not palpable. However, knowledge of these factors as potential barriers is crucial. Addressing and verbalizing them, together with clarifying the need for therapy to stabilize or improve visual function, may help to establish realistic expectations and thus improve adherence. Nevertheless, to date there are no outcome research studies exploring potential interventions to improve adherence in intravitreal therapy and thus therapeutic results. Interdisciplinary medical care, as typically carried out in diabetes care, may lead to improved results, when all care providers are aware of the necessary repetitive injections [70]. In addition, it appears that repetitive patient education is necessary. A recent analysis showed that after a few weeks, patients do not remember important details about their disease, the treatment, and possible complications despite thorough information having been provided and informed consent having been obtained [71]. This underlines the importance of repeatedly and regularly educating the patient, even during the course of the therapy.

### Limitations and the need for systematic research

There are limitations of our study. The analysis of factors associated with non-adherence has the inherent difficulty of being biased. Data may not be available at the point when non-adherence occurs, and in chronic (chorio-)retinal diseases, reasons for non-adherence may change or develop during the course of treatment. It is possible that practitioners do not allow insights that might make them appear in an unfavorable light. Hidden or insufficiently researched factors are not and cannot be found even in a meta-analysis. In this review, we analyzed studies with quantitative data, e.g., statistical calculations and association analysis, as well as studies with qualitative data, e.g., from patient interviews. Factors from all dimensions as defined by the WHO were identified in quantitative or qualitative analysis. For some factors, such as higher age, development of visual acuity, or greater distance to the treatment center, there was consistent and quantitative evidence in multiple studies. Some factors, however, were only identified in qualitative analysis, such as fear of adverse events or pain, or treatment burden.

Most of the studies analyzed patients with age-related macular degeneration, while only a few studies included patients with diabetic macular edema (DME) or retinal vein occlusion as subgroups. Although patients with DME seem to be more prone to become non-adherent, there is insufficient quantitative data to identify significant attributable factors [16, 17]. One study found that the reasons for non-adherence between patients with DME and nAMD were similar, although a significantly higher proportion of patients with DME gave “no explanation” at all for non-adherence in a telephone interview [16]. As patients with DME are younger in average than nAMD patients, conflicts with work or other appointments may present a greater barrier in this population. In addition, the minority of the overall healthcare visits (mean of 29 visit days per year) were due to eye care-related visit days, indicating the complex comorbidity profile and their care in diabetic patients with DME [72].

The majority of the included studies were retrospective, and the study designs varied with regard to the time of data extraction, time and mode of interviews, or even definition of non-adherence (see Table 1). Until now there has been no uniform definition of non-adherence or non-persistence which has contributed further to divergent findings. The most identified publications were from Europe, especially from Germany; thus, results are not directly transferable to other healthcare systems. Healthcare systems themselves differ considerably from one other, and this may have led to different treatment adherence [7], i.e., between European countries, or to different selection of patients receiving therapy. This can be seen in data from the AURA study: In the UK, patients are followed by a strict control and treatment regime, while treatment in other European countries, such as Germany, followed more individual and variable treatment plans. This, however, resulted in a significant lower number of OCT scans and IVT treatments in Germany, and clinical results were inferior to those in the UK [7]. Endeavors have been made to establish methods for the early detection of potential barriers, e.g., by early implementation of questionnaires in the management protocol [73]. However, it still remains to be evaluated whether this knowledge can be transferred into daily care and improve adherence and clinical results under everyday conditions.

The field of non-persistence seems to be a subject that is still completely underexposed. Of course, it is a very special problem to assess patients and their parameters which do not even appear in the routine. The deceased can no longer be interviewed; others with non-persistence may also be more critical of scientific surveys. Nevertheless, the knowledge of the poor outcome in the spontaneous course of the disease gives an idea of how important it is to avoid the termination of a necessary therapy as the maximum form of undertreatment.

### Knowledge of potential barriers and addressing them is crucial for long-term treatment success

In conclusion, this systematic review summarizes potential factors leading to non-adherence and non-persistence in intravitreal therapy. While modifiable factors may be addressed directly, knowledge and verbalization of unmodifiable factors still may be required in order to improve the attention of both the caring physician and the patient. In order to overcome the relative neglect of determinants and facilitate better patient support, a stronger commitment and coordinated action is needed from healthcare professionals and policy-makers. It seems that a further decrease in new cases of blindness will then be possible [74–77].

## Electronic supplementary material

ESM 1 (PDF 42 kb)

ESM 2 (PDF 87 kb)
