# Supplementary material for: Systematic review: non-adherence and non-persistence in intravitreal treatment
Source: Graefes Arch Clin Exp Ophthalmol. 2020 Jun 22;258(10):2077–90. doi: 10.1007/s00417-020-04798-2 (PMC7550304; doi:10.1007/s00417-020-04798-2)
Supplement: Supplementary file 1 — (PDF 42 kb) [file 417_2020_4798_MOESM1_ESM.pdf]

Systematic Review: non-adherence and non-persistence in intravitreal treatment

Graefe's Archive for Clinical and Experimental Ophthalmology

Christoph Ehlken, Focke Ziemssen, Nicole Eter, Ines Lanzl, Hakan Kaymak, Albrecht Lommatzsch and Alexander K. Schuster

Corresponding author:

Dr. med. Christoph Ehlken, F.E.B.O.
Klinik für Ophthalmologie des UKSH, Campus Kiel
christoph.ehlken@uksh.de

Suppl. 1: Complete list of search keywords from EMBASE and Medline (via PubMed) literature research

((('wet macular degeneration'/exp OR 'age related macular degeneration'/exp OR diabet* NEAR/3 macula* OR ('dme':ti AND macula*) OR (('retina*' NEAR/5 'venous') AND (venous NEAR/5 'occlu*')) OR 'retina* vein*' NEAR/5 'occlu*' OR 'diabetic macular edema'/exp OR 'retina vein occlusion'/exp OR 'central retina vein occlusion'/exp OR 'branch retinal vein occlusion'/exp) OR (exudat* OR wet OR neovascular*) NEAR/4 'age-related macular degeneration' OR (exudat* OR wet OR neovascular*) NEAR/4 'age related macular degeneration' OR (exudat* OR wet OR neovascular*) NEAR/4 'agerelated macular degeneration') AND (('patient compliance'/exp OR 'treatment refusal'/exp OR 'medication compliance'/exp OR 'patient dropout'/exp OR (complian* OR noncomplian* OR adhere* OR dropout* OR termination OR persistence) OR (nonadheren* OR nonpersistence OR 'healthcare constraint*' OR 'patient s perspective*' OR 'patients perspective*' OR barriers) OR ('treatment satisfaction*' OR discontinu* OR 'disease perception*' OR 'patient preference*')) OR (('coping':ab,ti OR 'comorbidit*':ab,ti OR 'pain':ab,ti) OR 'coping behavior'/exp OR 'comorbidity'/exp OR 'pain'/exp)) AND (((('aflibercept*':ti OR 'vegf trap*':ti OR 'eylea':ti OR 'eylear':ti OR 'eyleatm':ti OR 'ranibizumab*':ti OR 'lucentis*':ti) OR ('bevacizumab':ti OR 'avastin':ti OR 'avastine':ti OR 'avastinr':ti OR 'avastintm':ti) OR ('dexametason*':ti OR 'dexamthason*':ti OR 'ozurdex*':ti OR 'triamcinolon*':ti OR 'fluocinolon*':ti) OR 'pegaptanib*':ti OR 'aflibercept'/exp/mj OR 'bevacizumab'/exp/mj OR 'pegaptanib'/exp/mj OR 'ranibizumab'/exp/mj OR 'dexamethasone'/exp/mj OR 'triamcinolone'/exp/mj OR 'triamcinolone acetonide'/exp/mj OR 'triamcinolone acetate'/exp/mj OR 'triamcinolone diacetate'/exp/mj OR 'triamcinolone hexacetonide'/exp/mj OR 'dexamethasone acetate'/exp/mj OR 'dexamethasone cipecilate'/exp/mj OR 'dexamethasone isonicotinate'/exp/mj OR 'fluocinolone'/exp/mj OR 'fluocinolone acetonide'/exp/mj) OR ('anti vegf' OR antivegf) OR 'vegf':ti OR 'intravitreal*':ti OR 'intravitreal drug administration'/exp/mj) OR ('vascular endothelial growth factor*':ab,ti OR 'dexamethason*':ti))) NOT (((('wet macular degeneration'/exp OR 'age related macular degeneration'/exp OR diabet* NEAR/3 macula* OR ('dme':ti AND macula*) OR (('retina*' NEAR/5 'venous') AND (venous NEAR/5 'occlu*')) OR 'retina* vein*' NEAR/5 'occlu*' OR 'diabetic macular edema'/exp OR 'retina vein occlusion'/exp OR 'central retina vein occlusion'/exp OR 'branch retinal vein occlusion'/exp) OR (exudat* OR wet OR neovascular*) NEAR/4 'age-related macular

degeneration' OR (exudat* OR wet OR neovascular*) NEAR/4 'age related macular degeneration' OR (exudat* OR wet OR neovascular*) NEAR/4 'agerelated macular degeneration') AND (('patient compliance'/exp OR 'treatment refusal'/exp OR 'medication compliance'/exp OR 'patient dropout'/exp OR (complian* OR noncomplian* OR adhere* OR dropout* OR termination OR persistence) OR (nonadheren* OR nonpersistence OR 'healthcare constraint*' OR 'patient s perspective*' OR 'patients perspective*' OR barriers) OR ('treatment satisfaction*' OR discontinu* OR 'disease perception*' OR 'patient preference*')) OR (('coping':ab,ti OR 'comorbidit*':ab,ti OR 'pain':ab,ti) OR 'coping behavior'/exp OR 'comorbidity'/exp OR 'pain'/exp)) AND (((('aflibercept*':ti OR 'vegf trap*':ti OR 'eylea':ti OR 'eylear':ti OR 'eyleatm':ti OR 'ranibizumab*':ti OR 'lucentis*':ti) OR ('bevacizumab':ti OR 'avastin':ti OR 'avastine':ti OR 'avastinr':ti OR 'avastintm':ti) OR ('dexametason*':ti OR 'dexamthason*':ti OR 'ozurdex*':ti OR 'triamcinolon*':ti OR 'fluocinolon*':ti) OR 'pegaptanib*':ti OR 'aflibercept'/exp/mj OR 'bevacizumab'/exp/mj OR 'pegaptanib'/exp/mj OR 'ranibizumab'/exp/mj OR 'dexamethasone'/exp/mj OR 'triamcinolone'/exp/mj OR 'triamcinolone acetonide'/exp/mj OR 'triamcinolone acetate'/exp/mj OR 'triamcinolone diacetate'/exp/mj OR 'triamcinolone hexacetonide'/exp/mj OR 'dexamethasone acetate'/exp/mj OR 'dexamethasone cipecilate'/exp/mj OR 'dexamethasone isonicotinate'/exp/mj OR 'fluocinolone'/exp/mj OR 'fluocinolone acetonide'/exp/mj) OR ('anti vegf' OR antivegf) OR 'vegf':ti OR 'intravitreal*':ti OR 'intravitreal drug administration'/exp/mj) OR ('vascular endothelial growth factor*':ab,ti OR 'dexamethason*':ti))) AND ('review'/it OR 'short survey'/it))
