# Supplementary material for: Systematic review: non-adherence and non-persistence in intravitreal treatment
Source: Graefes Arch Clin Exp Ophthalmol. 2020 Jun 22;258(10):2077–90. doi: 10.1007/s00417-020-04798-2 (PMC7550304; doi:10.1007/s00417-020-04798-2)
Supplement: Supplementary file 2 — (PDF 87 kb) [file 417_2020_4798_MOESM2_ESM.pdf]

Systematic Review: non-adherence and non-persistence in intravitreal treatment

Graefe's Archive for Clinical and Experimental Ophthalmology

Christoph Ehlken, Focke Ziemssen, Nicole Eter, Ines Lanzl, Hakan Kaymak, Albrecht Lommatzsch and Alexander K. Schuster

Corresponding author:

Dr. med. Christoph Ehlken, F.E.B.O.
Klinik für Ophthalmologie des UKSH, Campus Kiel
christoph.ehlken@uksh.de

Suppl. 2: Studies evaluating pain and its risk factors during or after IVT

First author	Study type	Data acquisition	Number of patients/eyes	Objective	Results
**Alattas[59]**	prospective, uncontrolled	questionnaire	56	comparison of bimanual lid retraction versus metal speculum	bimanual lid retraction was associated with less discomfort
**Chua[60]**	consecutive case series	questionnaire	100	comparison of expected discomfort (before IVT) versus actual discomfort	actual discomfort was significantly less than expected discomfort
**Cintra[61]**	prospective, randomized	VAS	60	comparison of different methods of anesthesia (topical, subconjunctival, peribulbar)	less pain with peribulbar anesthesia compared to topical and subconjunctival
**Cohen[62]**	Prospective, single-blinded, randomized	interview	57	preference of anesthesia: subconjunctival versus topical	88% preferred subconjunctival anesthesia over topical
**Gregori[48]**	randomized	Interview, examination	50	discomfort and safety of subconjunctival versus topical lidocaine	both methods equally effective, less ocular irritation after topical

**Gueler[63]**	prospective, non-randomized	VAS	70	pain experience during injection with 30-gauge versus 27-gauge needles	30-gauge more tolerable than 27-gauge
**Kaderli[64]**	prospective, randomized	interview	28	comparison of different methods of anesthesia (topical, subconjunctival)	better pain control with subconjunctival, procedure itself more painful and hemorrhage
**Karimi[46]**	prospective, randomized	VAS	1004	correlation of injection site with pain	superotemporal most painful, superonasal least painful
**Kozak[65]**	crossover and consecutive groups	VAS	28	comparison of different methods of anesthesia (topical, subconjunctival)	no difference in pain scores, more chemosis and hemorrhage with subconjunctival
**Lagstein[66]**	prospective, randomized, double-blind	rating scale	39	comparison of pain under IVT with or without prior application of apraclonidine	prior application of topical apraclonidine significantly reduced pain
**Loureiro[47]**	cross-sectional, randomized	VAS	54	pain experience and intraocular during injection with 30-gauge versus 27-gauge needles	comparable pain/discomfort, lower IOP spikes after 27-gauge injection
**Makri[67]**	prospective, double-blinded, randomized	VAS, questionnaires	52	evaluation of analgesic effect of topical nepafenac	a single drop of topical nepafenac reduced pain at injection and up to 6 hours after
**Massamba[68]**	prospective interventional case series	VAS	112	evaluation of risk factors for pain	risk factors were: left eye, IVT location temporal superior quadrant. Not correlated: age, gender, number of injections, waiting time, history of cataract surgery
**Moisseiev[69]**	case series, matched control group	VAS	57	comparison of pain after Ozurdex versus bevacizumab	comparable discomfort for Ozurdex and bevaiczumab injections
**Rahimy[70]**	prospective, randomized, crossover	questionnaire, VAS	36 patients	comparison of bimanual lid retraction versus metal speculum	patients favored bimanual retraction over speculum
**Rifkin[71]**	prospective, randomized	VAS	60	comparison of different methods of anesthesia (tetracaine, proparacaine, TetraVisc)	pain is generally mild, no differences between topical medication
**Sanabria[72]**	prospective, randomized, double-masked	VAS	156	comparison of different pre- and post-IVT regimes (tetracaine+naphazoline versus lidocaine; tobramycin versus tobramycin+diclofenac)	effects of different regimes comparable
**Shiroma[73]**	prospective, randomized	VAS	260	comparison of five different concentrations of lidocaine gel	lidocaine drops effective and safe at concentrations from 2 to 12%

**Tailor[74]**	prospective, interventional case series	questionnaire	42	evaluation of distress during the different steps of the injection process	injection itself with highest percentage of distress; application and removal of drape and lid speculum caused >50% of distress
**Ulrich[75]**	prospective, double-masked, randomized	scale 0-3	120	evaluation of discomfort with and without application of a single drop of nepafenac before IVT	statistically less pain 6 hours after IVT with nepafenac, no statistically significant differences at 1 and 24 hours
**Van Asten[76]**	randomized, crossover	VAS	36	comparison of pain after use of 30-G and 33-G needles	pain comparable with both sizes, but potentially less scleral damage after 33-G needle (less reflux)
**Yau[77]**	prospective, randomized, double-blinded	VAS	93	comparison of 0.5% tetracaine hydrochloride with or without 4% lidocaine pledget versus 4% cocaine drops	no statistically significant differences

VAS: visual analog scale
